# FEASIBILITY OF SMART AMBIENT BRIGHT LIGHT (SABL) FOR NURSING HOMES RESIDENTS WITH DEMENTIA

**DOI:** 10.1093/geroni/igae098.3980

**Published:** 2024-12-31

**Authors:** Yo-Jen Liao, Ying-Ling Jao, Marie Boltz

**Affiliations:** The Pennsylvania State University, University Park, Pennsylvania, United States; The Pennsylvania State University, University Park, Pennsylvania, United States; The Pennsylvania State University, University Park, Pennsylvania, United States

## Abstract

Ambient bright lighting interventions have shown some positive benefits on nursing home (NH) residents with dementia. A feasible implementation approach and stakeholder buy-in have not been well evaluated. This study examined NH staff’s perspectives on the feasibility of implementing the Smart Ambient Bright Light (SABL). The SABL was installed in participants’ bedrooms and common areas in two NHs to automatically deliver bright light during the day and dim light during the night. This convergent mixed methods study enrolled 22 participants, including nursing, activity, maintenance, and administrative staff. Quantitative data were collected using the Acceptability of Intervention Measure, Feasibility of Intervention Measure, and Intervention Appropriateness Measure. Qualitative data were collected via semi-structured interviews and analyzed using content analysis. Quantitative results showed good feasibility (mean=4.4, SD=0.51), acceptability (mean=4.1, SD=0.9), and appropriateness (mean=4, SD=0.7) on a 1-5 scale. Qualitative results were categorized in installation, light brightness, automatic system, and resident responses categories. Maintenance staff reported that installing the SABL was relatively easy. Brightness wise, some reported that the lights were too bright while others thought it made people more awake during the day. Most staff reported that the lighting system was easy to use while others preferred manual control to better accommodate residents’ various routine. Some reported that residents showed improved neurobehavioral symptoms, sleep, mood, and engagement. Overall, staff perspectives on SABL were positive, but some improvements to the light brightness and automatic system are needed. Limitations on the facility’s building structure and lighting fixtures should be considered in future studies.

